# Supplementary material for: Visualizing the Spatial Distribution of Arctium lappa L. Root Components by MALDI-TOF Mass Spectrometry Imaging
Source: Foods. 2022 Dec 7;11(24):3957. doi: 10.3390/foods11243957 (PMC9778511; doi:10.3390/foods11243957)
Supplement: Supplementary file 1 [file foods-11-03957-s001.zip › foods-1997242-supplementary.pdf]

Supplementary Data

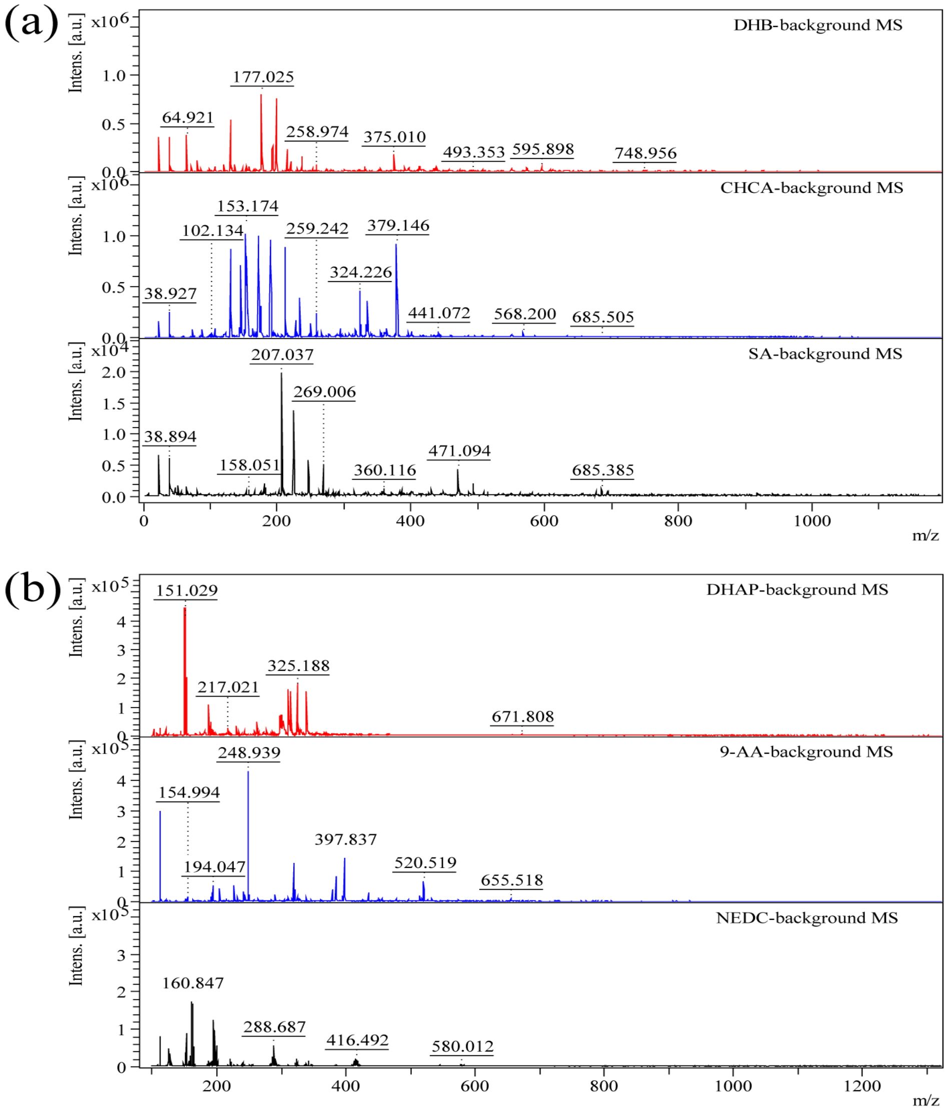

Figure S1. MALDI MS spectra of matrix background in positive (**a**) and negative (**b**) ion reflector mode

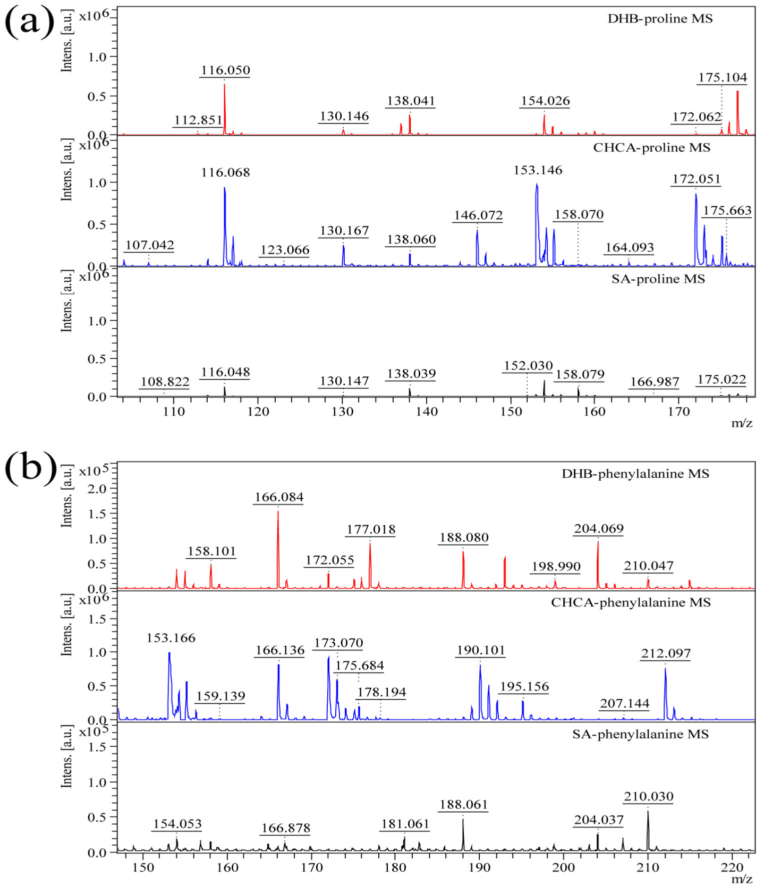

Figure S2. MALDI MS spectra of representative components using different matrices in positive ion reflector mode. Proline ($[M+H]^+$, m/z 116.1; $[M+Na]^+$, m/z 138.1; $[M+K]^+$, m/z 154.1) (**a**), and phenylalanine ($[M+H]^+$, m/z 166.1; $[M+Na]^+$, m/z 188.1; $[M+K]^+$, m/z 204.1) (**b**).

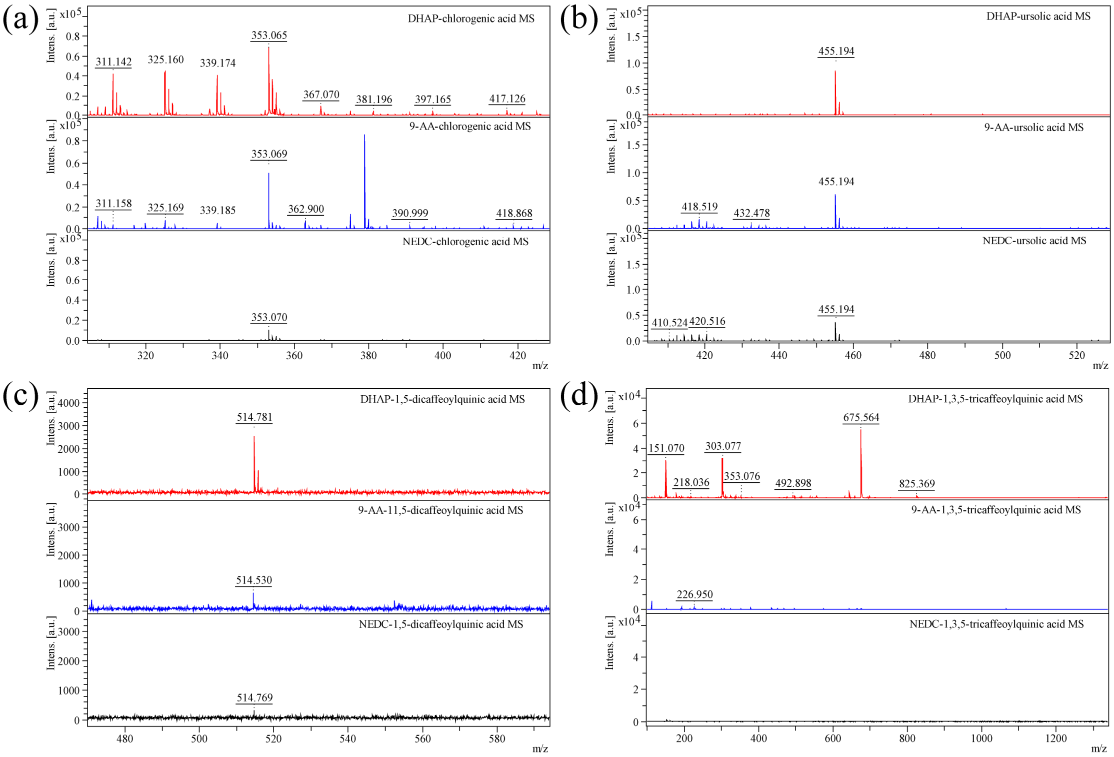

Figure S3. MALDI MS spectra of representative components using different matrices in negative ion reflector mode. Chlorogenic acid ([M−H]$^-$, m/z 353.1) (**a**), ursolic acid ([M−H]$^-$, m/z 455.2) (**b**), 1,5-dicaffeoylquinic acid ([M−2H]$^{2-}$, m/z 514.8) (**c**), 1,3,5-tricaffeoylquinic acid ([M−3H]$^{3-}$, m/z 675.6) (**d**).

(a)

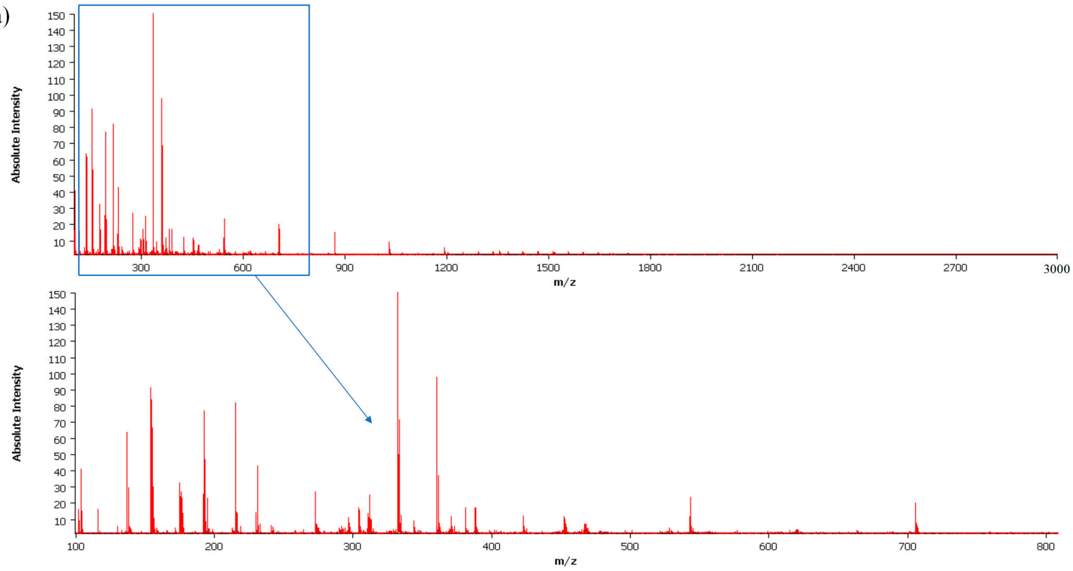

(b)

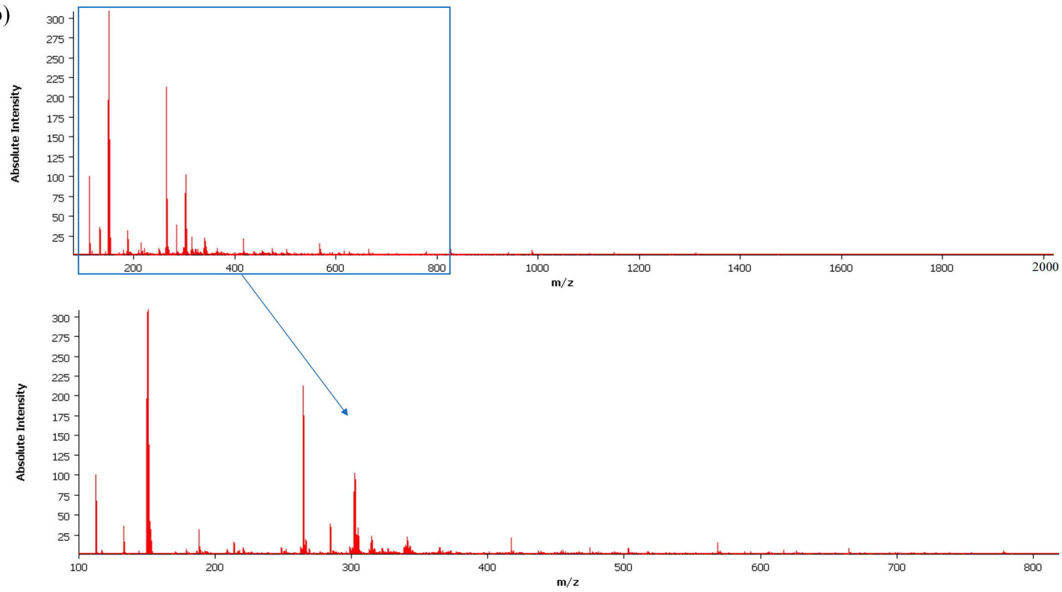

(c)

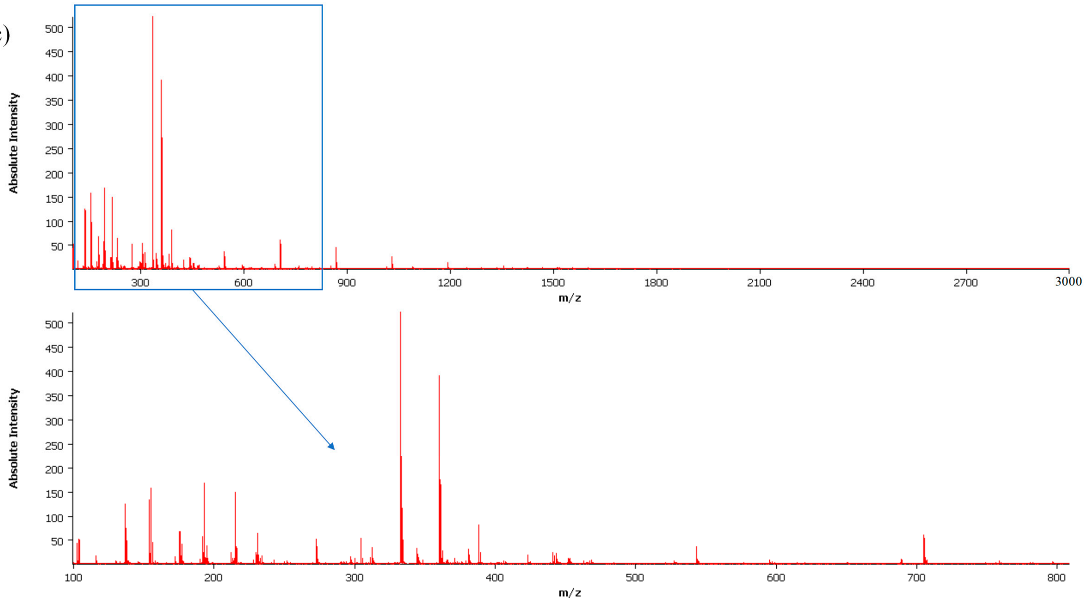

(d)

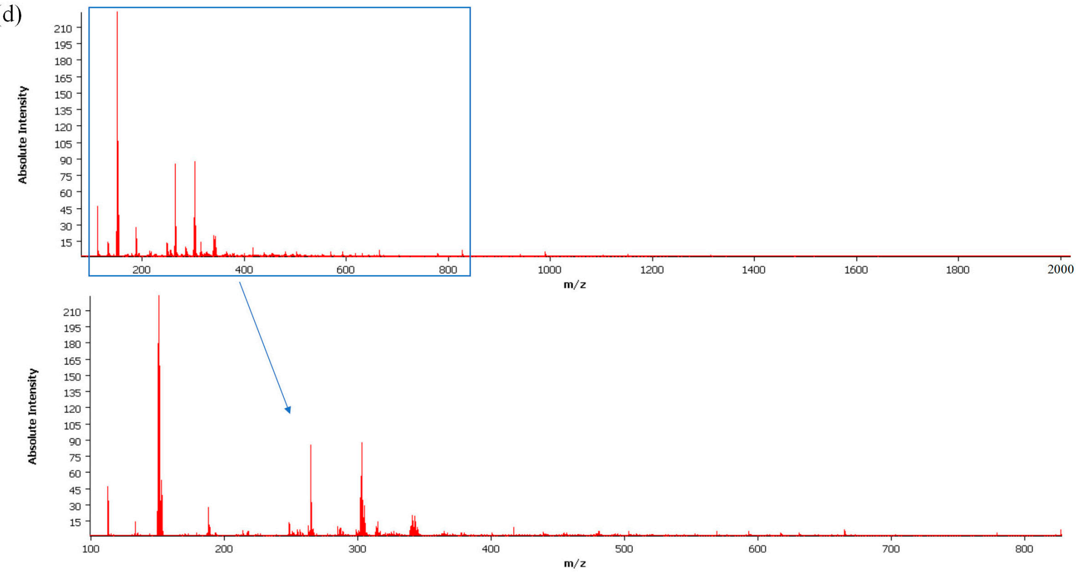

Figure S4. MALDI MS imaging fusion spectra of endogenous components in *A. lappa* using DHB in positive ion reflector mode and using DHAP in negative ion reflector mode. Endogenous components of *Baiji* variety using DHB in positive ion reflector mode (**a**) and using DHAP in negative ion reflector mode (**b**); Endogenous components of *Yanagawa-riso* variety using DHB in positive ion reflector mode (**c**), and using DHAP in negative ion reflector mode (**d**).

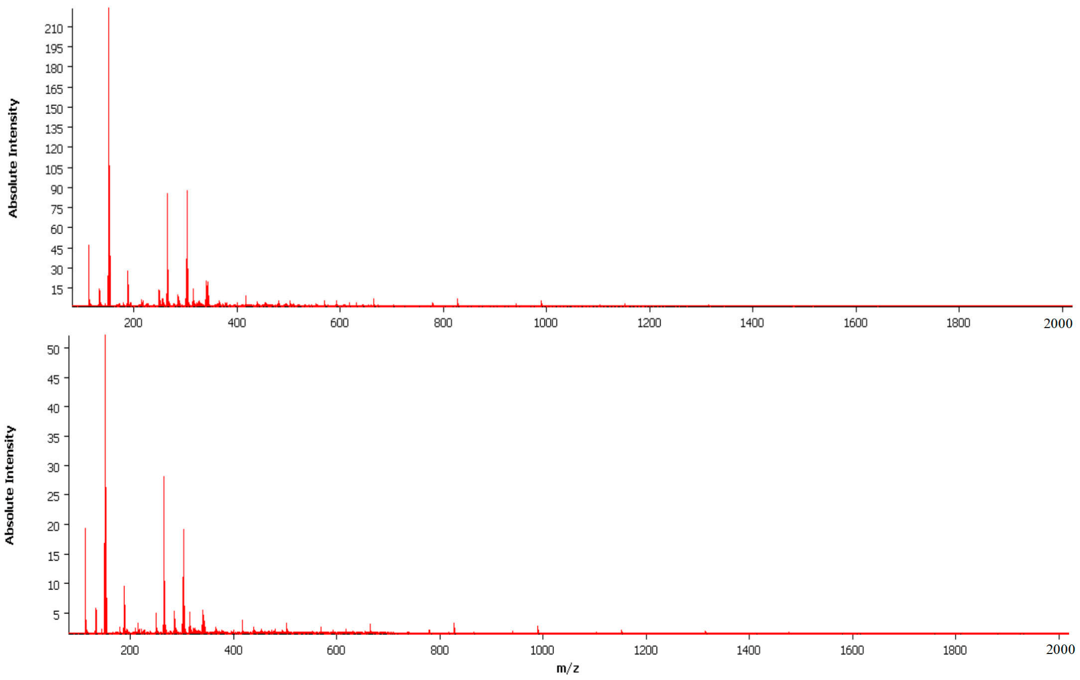

Figure S5. MALDI MS imaging fusion spectra of endogenous components in *Yanagawa-riso* variety of *A. lappa* using DHAP in negative ion reflector mode.

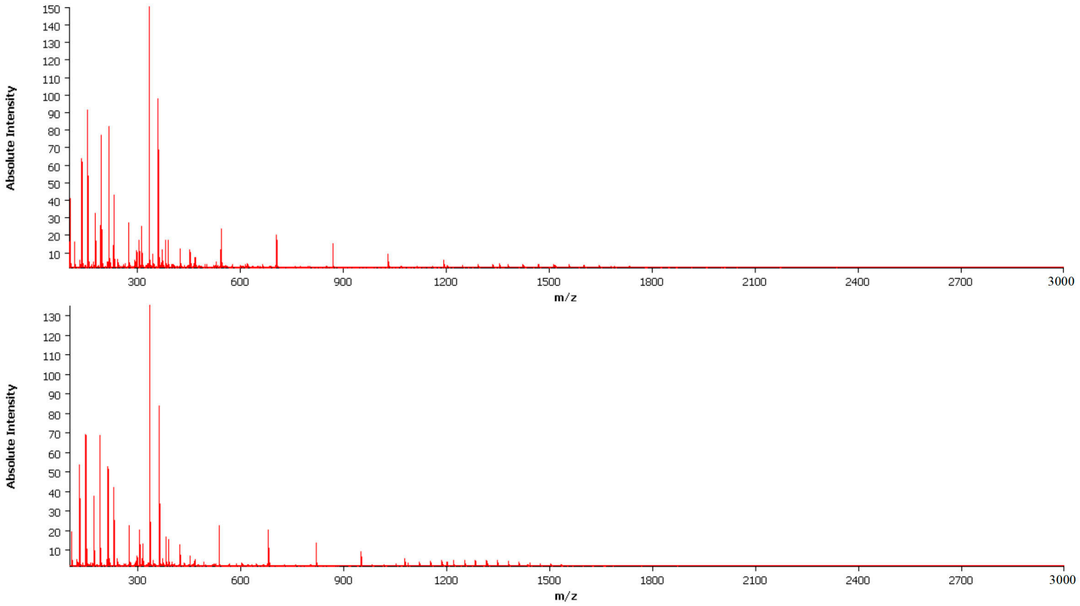

Figure S6. MALDI MS imaging fusion spectra of endogenous components in *Baiji* variety of *A. lappa* using DHB in positive ion reflector mode.
